# Recent development of antiSMASH and other computational approaches to mine secondary metabolite biosynthetic gene clusters

**DOI:** 10.1093/bib/bbx146

**Published:** 2017-11-03

**Authors:** Kai Blin, Hyun Uk Kim, Marnix H Medema, Tilmann Weber

**Keywords:** genome mining, biosynthetic gene cluster, antibiotics, secondary metabolites, natural products, antiSMASH

## Abstract

Many drugs are derived from small molecules produced by microorganisms and plants, so-called natural products. Natural products have diverse chemical structures, but the biosynthetic pathways producing those compounds are often organized as biosynthetic gene clusters (BGCs) and follow a highly conserved biosynthetic logic. This allows for the identification of core biosynthetic enzymes using genome mining strategies that are based on the sequence similarity of the involved enzymes/genes. However, mining for a variety of BGCs quickly approaches a complexity level where manual analyses are no longer possible and require the use of automated genome mining pipelines, such as the antiSMASH software. In this review, we discuss the principles underlying the predictions of antiSMASH and other tools and provide practical advice for their application. Furthermore, we discuss important caveats such as rule-based BGC detection, sequence and annotation quality and cluster boundary prediction, which all have to be considered while planning for, performing and analyzing the results of genome mining studies.

## Introduction

Most antibiotics, such as penicillin, erythromycin or tetracycline, and also other drugs like acarbose (anti-diabetic), artemisinin (anti-malarial), tacrolimus or cyclosporins (immunosuppressants) are so-called natural products either synthesized by or derived from microorganisms or plants [1]. As the biosynthetic pathways for such compounds are not directly related to growth and reproduction, these compounds are also referred to as ‘secondary metabolites’ or—in newer literature—‘specialized metabolites’. In bacteria and fungi, the genes required for the biosynthesis of these compounds are usually organized as biosynthetic gene clusters (BGCs). These clusters contain all genes required for the biosynthesis of precursors, assembly of the compound scaffold, modification of the compound scaffold (also referred to as ‘tailoring’) and often also resistance, export and regulation. This implies that the full pathway can easily be identified if the involvement of one of the genes in biosynthesis can be demonstrated. In plants, only some pathways are organized in BGCs [2]. For other pathways, the biosynthesis genes are scattered across the genome and thus require additional experimental data, such as co-expression analyses [3], for identification.

Soon after the first genes encoding natural product biosynthetic enzymes were identified, sequenced and analyzed, it became apparent that the sequences of the corresponding enzymes contain data of highly predictive quality, which can be used to infer key biosynthetic steps. For example, the core scaffolds of the products of canonical modular type I polyketide synthases (PKSs) can be predicted by combining several types of easy-to-obtain data: (a) the content and architecture of individual enzymatic domains within the megaenzymes, which are responsible for the assembly of the molecular scaffold and its modifications (e.g. reduction of the β-carbon), can be identified by using Hidden Markov model (HMM) profiles of such domains; (b) the individual acyl-CoA building blocks for each PKS module (e.g. malonyl-CoA versus methylmalonyl-CoA) can be inferred based on key residues in the active sites of the acyltransferase (AT) domains or by using phylogenetic classification; (c) the stereospecificity mediated by ketoreductase domains can be inferred by key amino acids in the active site motifs. These studies were the starting point in establishing genome mining for secondary metabolite BGCs as one of the recent key technologies in natural products research.

One of the first computational tools to make use of such predictions was the proprietary DECIPHER^Ⓡ^ search engine and database of the former company Ecopia [4] that was first published in 2003. Around the same time, the first publicly available tools were released. For example, SEARCHPKS automated the identification of enzymatic domains in PKSs [5] (for URLs to this and all following Web tools, please see Table 1). However, it took until 2009 for the first open-source genome mining pipelines CLUSEAN [29] and NP.searcher [21] to be published. In 2011, the first version of the open-source genome mining platform antiSMASH was released [30], which combined and extended the functionality of the previous tools and also offered a user-friendly Web interface. For the first time, it became possible for scientists without significant experience in computational biology to perform larger-scale genome mining studies on a free and public Web server. Since then, antiSMASH has been steadily extended [6, 7, 23, 30–33] and currently offers a broad collection of tools and databases for automated genome mining and comparative genomics for a wide variety of different classes of secondary metabolites. The antiSMASH analysis pipeline for bacterial genomes and the pipeline for fungal genomes (recently named ‘fungiSMASH’) are both based on the same codebase. antiSMASH and fungiSMASH use two different Web submission forms, each offering specific options. plantiSMASH [23] is a branch of antiSMASH that includes plant-specific functionality, such as plant-adapted HMM profiles and cluster detection logic, as well as support for coexpression analysis.

**Table 1:** URLs of Web servers, Web tools and databases referred to in the review

Tool	Functions	URL	Reference
antiSMASH 4	Genome mining	http://antismash.secondarymetabolites.org	[6]
	BGC analysis		
	Domain analysis		
antiSMASH database	BGC database	http://antismash-db.secondarymetabolites.org	[7]
ARTS	Genome mining	http://arts.ziemertlab.com	[8]
BAGEL 3	Genome mining	http://bagel.molgenrug.nl/	[9]
CASSIS	BGC boundary prediction	https://sbi.hki-jena.de/cassis/cassis.php	[10]
CRISPy-web	sgRNA design	http://crispy.secondarymetabolites.org	[11]
eSNaPD v2	Genome mining	http://esnapd2.rockefeller.edu	[12]
FunGeneClusterS	BGC boundary prediction	https://fungiminions.shinyapps.io/FunGeneClusterS	[13]
fungiSMASH	Genome mining	http://fungismash.secondarymetabolites.org	[6]
	BGC analysis		
	Domain analysis		
GNP	Metabolomics	http://magarveylab.ca/gnp	[14]
GRAPE/GARLIC	Genome mining	https://magarveylab.ca/gast/	[15, 16]
MIBiG	BGC database	http://mibig.secondarymetabolites.org	[17]
	reference data set		
NaPDoS	Genome mining	http://napdos.ucsd.edu	[18]
NORINE	Nonribosomal peptide database	http://bioinfo.lifl.fr/NRP	[19, 20]
NP.searcher	Genome mining	http://dna.sherman.lsi.umich.edu/	[21]
	Domain analysis		
NRPSpredictor	Domain analysis	http://nrps.informatik.uni-tuebingen.de	[22]
plantiSMASH	Genome mining	http://plantismash.secondarymetabolites.org	[23]
	BGC analysis		
PRISM 3	Genome mining	http://magarveylab.ca/prism	[24]
	BGC analysis		
	Domain analysis		
RODEO	Genome mining	http://www.ripprodeo.org	[25]
	RiPP analysis		
(SEARCHPKS)/SBSPKS v2	Domain analysis	http://202.54.226.228/∼pksdb/sbspks_updated/master.html	[26]
	BGC database		
Smiles2Monomers	Retro-biosynthetic monomer prediction	http://bioinfo.lifl.fr/norine/smiles2monomers.jsp	[27]
SMURF	Genome mining	http://www.jcvi.org/smurf	[28]

In addition to antiSMASH, other noteworthy tools have also been developed and made available: SMURF [28] offers mining for fungal PKS, nonribosomal peptide synthetase (NRPS) and terpenoid gene clusters; the PRISM tool [24, 34, 35] offers genome mining functionality with a strong focus on predicting chemical structures of the biosynthetic pathways. PRISM is closely connected to the ‘Genomes-to-Natural Products platform (GNP)’ [14] that matches such predictions with MS/MS data, and to the GRAPE/GARLIC tools [15, 16], which match the predictions to chemical databases. For a comprehensive review describing the history and progress of secondary metabolite genome mining, along with many examples of compounds and BGCs that were identified using genome mining approaches, please see [36].

**Figure 1:**
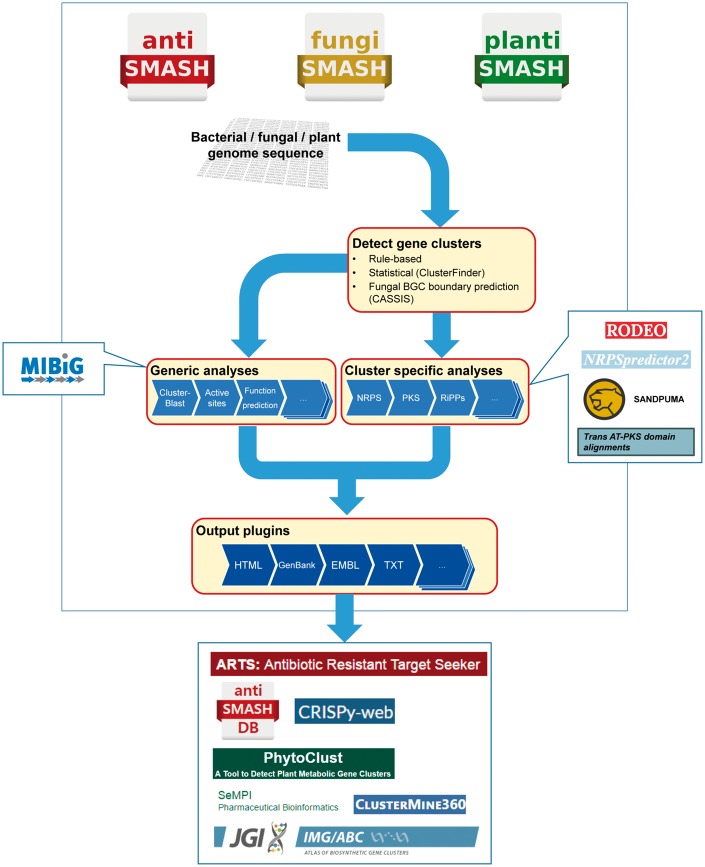
General workflow of an antiSMASH analysis of bacterial, fungal and plant genomes. Computational resources in the left and right boxes have been integrated with antiSMASH 4 for enhanced genome mining performance, whereas those in the box in the bottom correspond to third-party applications that use antiSMASH for the detection of BGCs.

In this review, we will focus on the general computational approaches to study secondary metabolite biosynthesis and how these are integrated into the current antiSMASH framework (Figure 1). Finally, we will give practical advice for preparing and interpreting genome mining data. Although we focus on antiSMASH as an example, the issues discussed are applicable to natural product genome mining in general, and hence are equally relevant when using other tools. Comprehensive user guides for antiSMASH can be found online (http://docs.antismash.secondarymetabolites.org/using_antismash/) and in [37–39]. For comprehensive reviews on the different genome mining tools and databases on secondary metabolites, the reader is referred to [40–43].

## Principles of predicting secondary metabolite biosynthesis

To predict secondary metabolite biosynthesis pathways, genome mining approaches commonly start out by identifying conserved biosynthetic genes. Their gene products are subsequently analyzed to gain information about their putative function in biosynthesis and sometimes their substrate specificity.

To identify conserved biosynthetic genes, it is necessary to have gene annotations available on the genome of interest. Formats such as NCBI’s GenBank or EBI’s EMBL contain both DNA sequence and gene annotations. GFF3 files can be used to carry the annotations for sequences in FASTA format. antiSMASH accepts input data in all of these formats. If no gene annotations are available, antiSMASH will run a gene finding tool. For the bacterial version, this is Prodigal [44]. For fungal and plant genomes, antiSMASH uses GlimmerHMM [45].

In the next step, BGCs are identified based on core enzymes involved in the biosynthesis of secondary metabolites. Functionally related proteins frequently share common patterns of amino acids. Using profile-based methods like position-specific scoring matrices to identify these patterns seems intuitive. HMMs are probabilistic models of linear sequences that provide an algorithmic approach to interpret the scores obtained from the scoring matrix. Profile HMMs (pHMMs) are HMMs designed to represent multiple sequence alignments, including matches, insertions and deletions. The most commonly used tool around pHMMs in biology is HMMer [46]. Many profile databases such as PFAM [47] and TIGRFAMs [48] provide downloadable profiles compatible with HMMer. antiSMASH uses pHMMs with profiles specific to conserved core enzymes of secondary metabolite biosynthesis pathways to run its profile-based BGC detection. Once the core enzymes have been identified, antiSMASH compares co-located core genes with a set of manually curated BGC cluster rules. These rules comprise Boolean logic regarding domain presence/absence within either a gene or a genomic region of interest. For example, BGCs encoding nonribosomally synthesized peptides (such as the antibiotic vancomycin) can be unambiguously identified if the sequence to be analyzed contains genes encoding proteins that have a combination of one or multiple Condensation, Adenylation (A) and Peptidyl Carrier Protein domains. ‘Negative’ models are also used to discard false positives, e.g. protein sequences that achieve higher scores for profiles of fatty acid synthases (which are homologous to PKSs) than for profiles of PKSs will not lead to the identification of a polyketide BGC. The 2017 version of antiSMASH (version 4) [6] uses such rules for 45 different types/classes of secondary metabolites (Table 2A). The cluster rules are stored in a tab-delimited text file, which can be easily edited to add custom types of gene clusters. Similar rule-based strategies are also used by many other secondary metabolite genome mining tools, such as PRISM [24], SMURF [28] and BAGEL [9].

**Table 2:** A: BGC types detectable by pHMM-based rules with antiSMASH, PRISM and SMURF. B: Rule-independent methods to detect BGCs

A: Rule-based detection of gene clusters^a^			
BGC-type	antiSMASH	PRISM/RiPP PRISM	SMURF
Aminocoumarins	X	X	
Aminoglycosides/ aminocyclitols	X		
Antimetabolites		X	
Aryl polyenes	X	X	
Autoinducing peptide		X	
Bacteriocins	X		
Beta-lactams	X	X	
Bottromycin	X	X	
Butyrolactones	X	X	
ClusterFinder fatty acid	X		
ClusterFinder saccharide	X		
ComX		X	
Cyanobactins	X	X	
Ectoines	X	X	
Furan	X	X	
Fused (pheganomycin-like)	X		
Glycocin	X	X	
Head-to-tail cyclized peptide	X	X	
Homoserine lactone	X	X	
Indoles	X	X	
Ladderane lipids	X	X	
Lantipeptides class I	X	X	
Lantipeptides class II	X	X	
Lantipeptides class III/IV	X	X	
Lasso peptide	X	X	
Linaridin	X	X	
Linear azol(in)e-containing	X	X	
Melanins	X	X	
Microcin	X		
Microviridin	X	X	
Nonribosomal peptides	X	X	X
Nucleosides	X		
Oligosaccharide	X		
Other (unusual) PKS	X		
Others	X		
Phenazine	X	X	
Phosphoglycolipids	X	X	
Phosphonate	X	X	
Polyunsaturated fatty acids	X		
Prochlorosin		X	
Proteusin	X	X	
Sactipeptide	X	X	
Non-NRP siderophores	X		
Streptide		X	
Terpene	X		X
Thiopeptides	X	X	
Thioviridamide		X	
Trans-AT type I PKS	X	X	
Trifolitoxin		X	
Type I PKS	X	X	X
Type II PKS	X	X	
Type III PKS	X	X	
YM-216391		X	

**Table 2:** (continued)

B: Rule-independent methods			
Method	Principle	Implemented in	References
ClusterFinder	HMM-based classification of which PFAM domains are likely to be found inside or outside a BGC	antiSMASH	[6, 49]
EvoMining	Phylogenomic identification of enzymes with expanded substrate spectrum; such enzymes are often found in BGCs	EvoMining	[50]
Resistance gene-based mining	Identification of potential antibiotic resistance genes; often such genes are part of BGCs to provide self-protection of the producing organism	ARTS	[8]

^a^For details on the pHMM’s and specific rules used by the different genome mining programs, please consult the original publications of antiSMASH [6, 32], PRISM [24, 34] or SMURF [28].

Alternatively, a probabilistic method to detect potential secondary metabolite BGCs can be selected in antiSMASH that uses the ClusterFinder algorithm [49]. Rather than using explicit rules requiring specific enzymes to be present for a particular class of BGCs, ClusterFinder is based on a model built from a training set of PFAM domains found in BGCs and non-BGC regions. Given this model and a genome of interest with annotated PFAM domains, ClusterFinder then calculates the probability of a stretch of observed PFAM domains to constitute a BGC. In regions where this probability is higher than the configurable threshold, a BGC is predicted.

For BGCs encoding NRPS, PKS, terpene or ribosomally synthesized and posttranslationally modified peptides (RiPPs), it is possible to perform some additional analyses to predict further details, such as substrate specificities or product cyclization patterns. To this end, it is sometimes necessary to classify proteins or domains that share a high overall sequence similarity. The differences between the functional classes (e.g. different substrate specificities) are determined by a small number of key amino acids. Sequence-alignment-based methods such as BLAST and profile-based methods like HMMer tend to perform poorly in these cases. As both kinds of methods are designed to score overall sequence similarities, they—by design—gloss over the few key differences. In such cases, more complex algorithms can be used. Support vector machines (SVMs) are a machine learning approach that uses supervised learning to create nonprobabilistic binary linear classifiers. SVMs classify data points encoded in multidimensional feature vectors by a maximum margin hyperplane. Compared with other machine learning methods such as artificial neural networks, the construction of the SVM hyperplane allows for gaining some insight over which of the input parameters contribute most to the solution.

For the multimodular enzymes involved in NRPS biosynthesis, antiSMASH uses the recently published SANDPUMA tool [51] to predict the substrates of A domains. Knowledge of these substrates and the order of the A domains are then used to predict the backbone structure of the NRPS product. SANDPUMA internally uses a combination of pHMMs and SVMs to obtain the best possible A domain substrate predictions. In RiPP clusters that encode the biosynthesis of, e.g., lanthi-, lasso-, sacti- and thiopeptides, identifying the precursor peptide is key to predicting the cluster product. Here, antiSMASH scores putative precursor peptides using the recently published RODEO tool [25], as well as some custom pHMMs. RODEO also uses both pHMMs and SVMs internally to identify precursor peptides. Tailoring enzymes that further modify the RiPP are also identified using pHMMs.

Phylogenetic analysis assists with the classification of enzymes in Clusters of Orthologous Groups and the calculation of phylogenetic distances of genes/enzyme sequences of interest to characterized reference sequences. Multiple methods exist to construct phylogenetic trees based on multiple sequence alignments. Depending on the desired output tree characteristics, the number of input sequences and other constraints, the most appropriate method should be chosen. A popular algorithm among the distance-matrix-based methods is the Neighbor-Joining algorithm, which uses bottom-up clustering to create the tree. Neighbor-Joining is a comparatively fast method, but the correctness of the tree depends on the accuracy and additivity of the underlying distance matrix. Maximum parsimony methods try to identify the tree that uses the smallest number of evolution events to explain the observed sequence data. While maximum parsimony algorithms build accurate trees, their computation tends to be relatively slow compared with distance matrix-based methods. Maximum likelihood methods use probability distributions to assess the likelihood of a given phylogenetic tree according to a substitution model. This method unfortunately has a high complexity for computing the optimal tree. Many current tools use a combination of methods. The popular software FastTree [52] first builds rough Neighbor-Joining trees and then refines them using a maximum likelihood scoring of the trees generated in the first pass.

In antiSMASH, phylogenetic methods are used in many places. For NRPS clusters, SANDPUMA includes a phylogenetic analysis in the PrediCAT step. A modified version of PrediCAT trained on a recently released data set [53] is also used in terpenoid clusters to further classify terpene synthases. Noncore biosynthetic genes in a BGC are assigned to ‘secondary metabolite clusters of orthologous groups’, for which phylogenies are reconstructed.

In addition to BGC type-dependent analyses, antiSMASH also includes general tools providing information on all cluster types. The built-in ClusterBlast module [30] considers the similarity of individual gene products as well as their genomic arrangement. ClusterBlast contains a comprehensive database of all predicted BGCs from publicly available genomes that is searched to identify organisms containing similar BGCs. The same algorithm is used in the ‘SubClusterBlast’ module to identify operons/sets of genes in the query BGC that code for enzymes involved in the biosynthesis of common precursors, for example the nonproteinogenic amino acid 3, 5-dihydroxy-phenylglycine present in some types, or NRPS clusters such as the vancomycin-family glycopeptides. Finally, this strategy is also used to search the Minimum Information on Biosynthetic Gene cluster (MIBiG) [17] data set with the ‘KnownClusterBlast’ function to provide information about related and well-characterized gene clusters. This function can also be used to perform a sequence-based dereplication, i.e. the identification of gene clusters that code for already known products.

## ‘Linked’ tools and resources

A general challenge when using comparative approaches to study BGCs is the varying quality of annotation in public sequence databases. Some BGCs that have been extensively studied experimentally are well annotated, whereas others—mostly identified in high-throughput sequencing efforts—were only annotated using standard genome annotation pipelines that do not provide specific annotations of secondary metabolite BGCs. Therefore, a community effort has been established to define a ‘MIBiG’ standard [17] and provide a standardized repository for BGCs that have been experimentally connected to their biosynthetic products. The MIBiG repository currently (as of April 2017) contains 1396 entries of BGCs that are validated to code for a specific biosynthetic pathway. Within this set, 396 of the entries contain comprehensive manually curated annotations of the specific features of the gene clusters, which were provided by the specialists that studied these respective BGCs. This collection now serves as a reference data set for a wide variety of applications and the validation of novel computational tools.

In addition to analyses integrated into antiSMASH, the annotation generated by antiSMASH can also be useful as a starting point for further downstream analyses. Therefore, antiSMASH 4 provides an application programming interface that allows third-party software to access antiSMASH annotation for further processing. Examples of such tools are the ‘Antibiotic Resistant Target Seeker ARTS’ [8], which predicts potential targets of antibiotics and uses the annotation provided by antiSMASH to mine for BGCs and CRISpy-web [11], a Web tool that allows user-friendly design of single guide RNAs (sgRNAs) for CRISPR applications on nonmodel organisms.

antiSMASH is a comprehensive genome mining platform, but only provides information on individually submitted genomes and does not offer any integrated search functionality. Therefore, in 2016, the antiSMASH platform was extended with a database containing precomputed antiSMASH annotation on >3900 finished high-quality bacterial genome sequences [7]. Using the Web interface, it is possible to browse secondary metabolite clusters by BGC type or taxonomy of the producer organism. Additionally, custom queries can be constructed using an interactive query builder. This makes it possible to answer research questions such as ‘which clusters of type NRPS contain A domains that select for the nonproteinogenic amino acid 3, 5-dihydroxy-phenylglycine?’ or ‘what BGCs of type RiPP exist in the genus *Streptomyces* that are not lanthipeptides?’. The results are displayed in the same antiSMASH Web format. They can also be exported in various file formats that allow further processing in other bioinformatics tools.

## Considerations and caveats for computational genome mining

### You can only find what you are looking for…

Most genome mining platforms, including antiSMASH (with default search options), SMURF [28] and PRISM [24, 34], use a rule-based approach to define what is annotated as a secondary metabolite BGC. These rules are derived from existing knowledge about key biosynthetic steps/principles, which require the activity of individual or combinations of specific enzymes. The genes encoding these are also often referred to as ‘core’ genes and used as anchors or probes to screen the genomic data of interest. While this method is highly sensitive and precise for identifying biosynthesis genes for many classes of secondary metabolites, such as polyketides, or nonribosomally synthesized peptides, it of course implies that only pathways for which rules are implemented in the mining software can be detected; all pathways that may use unknown or unrelated alternative enzymes will be missed.

As an extension to the rule-based genome mining, antiSMASH optionally provides the possibility to use the ‘ClusterFinder’ method [49]. This algorithm can identify BGCs that are not detected by the expert-generated rule sets described above. However, it should be noted that this method still has some bias, as the source data used to train the HMM determining whether a gene product likely belongs to a BGC are also based on the currently known pathways.

To address these limitations, alternative methods are under development to access the ‘biosynthetic dark matter’ and identify novel pathways and enzymes. One promising approach is ‘EvoMining’ [50], which is based on the observation that biosynthetic enzymes and/or resistance genes often evolved by duplication and divergence of primary metabolism enzymes. By detecting divergences in phylogenetic trees of enzymes from the core metabolism shared between many bacterial species, this method can identify enzymes that have likely been repurposed for secondary metabolite biosynthesis [50] or resistance [8]. Once novel pathways have been identified using such methods and experimentally validated, the newly obtained knowledge on the involved enzymes is of course used to refine and extend the rule-based mining methods.

### The quality of input data is important for getting reliable results

One important aspect to be considered when mining genomic data for BGCs using antiSMASH or alternative pipelines, such as PRISM [24, 34], SMURF [28] and ClusterFinder [49], is the quality of the sequence data that is to be analyzed. All these tools use either rule-based or statistical approaches to identify the BGCs involved in secondary metabolism. Both methods require that the sequence data to be analyzed are not too fragmented and that the genes of a BGC are not scattered across different contigs in the assembly. Users should be particularly aware of potential quality issues when analyzing genome data generated with short-read sequencing technologies. Special care has to be taken when analyzing type I polyketide or NRPS-containing BGCs; both types of pathways involve large multimodular megaenzymes, whose gene sequences often are highly repetitive and therefore difficult to assemble purely based on short sequencing reads [54]. The same applies to metagenomic data; reliable identification of BGCs—which consist of several genes—is only possible on well-assembled data. Therefore, analyses on the public antiSMASH Web server are limited to sequences of over 1 kb length and the first 1000 contigs. Both limits can be deactivated in the stand-alone version of antiSMASH. To analyze highly fragmented short-read-based assemblies, pipelines focusing on the detection and analysis of individual core domains, such as NaPDos [18] or eSNaPD [12], should be considered. In general, phylogenomics-based approaches like the abovementioned or as used in EvoMining [50] are excellent alternatives for such fragmented data, as they base their predictions on single enzymes/genes instead of requiring the presence of complete or partial BGCs [55]. Therefore, we recommend first using these tools to identify ‘interesting’ sequence records in such bulk DNA data and then submitting only these records (provided they have the required sequence length) for an analysis with antiSMASH.

In addition, most algorithms predicting enzyme specificities rely on automatically generated alignments of the user-supplied input data with experimentally characterized ‘reference’ sequences to identify residues of the active sites or the substrate-binding pockets. Depending on the tool used to predict specificities, these alignments are generated using standard multiple sequence alignment software like ClustalW [56] or Muscle [57]. Alternatively, BLAST or HMMer are used to match the query with a custom reference database. Consequently, these tools are sensitive to sequencing errors if these errors occur in or near the active sites or binding pockets. In addition, the accuracy of such computer-generated, nonrefined alignments may suffer if the protein sequence of interest is too dissimilar to the reference data sets. In both cases, this can easily lead to incorrect specificity predictions.

In the case where users analyze annotated sequence data, which is uploaded as GenBank files or directly retrieved from the NCBI GenBank or RefSeq database, antiSMASH will only consider the annotated genes and not perform additional gene finding. This also implies that genes annotated as ‘pseudogenes’ are not considered for any prediction. This is noteworthy, as many modular PKS and NRPS gene calls that were generated with the NCBI PGAP [58] pipeline (which is used to annotate all microbial genomes in RefSeq [59]) were inaccurate and the intact genes were labelled as pseudogenes. This bug has been fixed for RefSeq 82, but users that downloaded earlier versions of RefSeq entries should be cautious. Many GenBank records that were annotated with affected versions of PGAP also suffer from this issue.

If users supply unannotated sequence data, antiSMASH uses the software prodigal [44] for bacterial genomes or GlimmerHMM [45] for fungal and plant sequences to automatically identify coding regions. The downstream genome analyses therefore depend on the accuracy of the automated gene finding, which can vary between different organisms and is also dependent on the sequence quality. If users supply annotated sequence data by uploading GenBank-formatted or FASTA+GFF3 files, antiSMASH uses these gene coordinates. If an annotated and high-quality genome sequence of an organism of interest is available, it is therefore advisable to use the preannotated data.

### Defining the extent of a secondary metabolite BGC

Predicting the boundaries of a BGC solely based on genomic data still remains challenging. For fungal BGCs, conserved binding sites of cluster-specific transcriptional regulators are good indicators to use in defining which genes are co-regulated. If the same regulator binding site is present near the core-genes of a cluster, they probably belong to the same biosynthetic pathway. This approach is used in the CASSIS tool [10], which was recently integrated into version 4 of antiSMASH [6]. In addition, fungal transcriptomics data can also be used to efficiently define the cluster boundaries [60], as implemented in the FunGeneClusterS application [13].

For bacterial sequences, such automated or semi-automated methods are unfortunately not (yet) well established. The presence or absence of BGCs is often strain specific [61, 62]. Comparing genomes between closely related species to identify which genes are highly conserved between these species and which are unique to the strain of interest can indicate the extent of BGCs. In antiSMASH, we have therefore chosen an ‘inclusive’ approach. Genes that are encoded within an empirically defined distance from conserved core genes of a BGC are displayed as a cluster. The distances were selected in a way that we would rather overpredict the distance, i.e. include genes in the gene cluster annotation that may belong to the gene cluster border region, than exclude genes that are part of the BGCs but are encoded outside this range from the core biosynthetic genes.

### Strategies to connect gene clusters to molecules

In the end, most users turn to antiSMASH or related tools to accomplish one of two goals: (1) to identify potentially new molecules that could be synthesized by the organism of study based on its genome, or (2) to identify genes involved in the biosynthesis of an already observed molecule. Specific strategies are available for each of these scenarios.

When trying to find out what kind of specialized metabolites an organism can produce based on its genome, the starting point is to go over each gene cluster in the genome in detail. First, comparisons with BGCs from MIBiG (in antiSMASH, this is done using the KnownClusterBlast module) will identify BGCs that are either closely or more distantly related to these reference clusters. To determine whether a BGC is likely to produce the exact same molecule, manual inspection is required. It should be checked that all key biosynthetic genes of the reference cluster are also found in the BGC of interest by studying the data of the MIBiG entry and related literature. If so, are any additional enzymes encoded in the BGC of interest that could encode chemical modifications not observed for the known molecule? If the BGC encodes PKSs or NRPSs, do the domain architectures and their corresponding predicted substrate specificities match to those of the known cluster? The answers to these questions will determine whether the BGC of interest is likely to encode the biosynthesis of: (a) the same molecule (all relevant genes ‘shared’ with high percent identity, and perfect alignment of chemistry predictions with the structure of the known molecule); (b) a potentially new variant of a known molecule (some enzyme-coding genes are cluster-specific, and/or some substrate specificities are different); (c) a new molecule within a known class of molecules (only a minority or small majority of the genes ‘shared’); or (d) an altogether unknown molecule (no significant similarities). Before it can be concluded that a molecule is unknown, it should be taken into account that some known natural products lack a described BGC; hence, some novel-looking BGCs may still encode the production of molecules for which the chemistry has been long known. For polyketides and nonribosomal peptides, these cases can be assessed with a retro-biosynthetic approach using tools like Smiles2Monomers [27] or GRAPE [15]. These tools predict the potential monomers of a given compound structure, for example derived from a compound database. In a second step, these compounds can be connected to BGCs by mapping the monomer predictions derived from the chemical structure to the monomer predictions derived from the analysis of BGCs. The latter predictions can be made using the antiSMASH database or tools like GARLIC [15]. For nonribosomal peptides, another option is to check for compounds with similar monomers in the NORINE database [19, 20]. antiSMASH provides the appropriate search links from the ‘detailed annotations’ sidebar. If no cluster-wide similarity is observed, it is in any case still a good idea to look for similarities to known clusters at a smaller scale: either per gene or per subcluster. antiSMASH offers functionalities to identify such similarities, using the SubClusterBlast feature and the gene-specific BLAST search of MIBiG [17]. This makes it possible to predict the presence of specific chemical moieties or chemical modifications to the molecule, which helps to prioritize the targets or to connect the gene cluster to a molecule observed in metabolomic data. Finally, looking for functional markers can greatly help in prioritizing BGCs, e.g. when the aim of the project is antibiotic discovery, one can look for both general and specific types of antibiotic resistance genes that are often encoded inside a BGC to provide natural self-resistance to the producer [8, 16].

Sometimes, the structure of a molecule has already been elucidated before a genome is sequenced or studied. In such a case, the aim of using antiSMASH or related tools is usually to identify the biosynthetic mechanism of the molecule of interest. If, chemically, the molecule is closely related to other known natural products for which the biosynthesis is known, one would usually be able to find either a single BGC or only a few BGCs with high similarity to the corresponding MIBiG reference cluster. However, this is often not the case. Then, the best strategy is to use ‘exclusion logic’ and step-by-step exclude BGCs that are unlikely to be involved in the biosynthesis of the molecule, thus gradually narrowing down the options to only one or a few gene clusters. First, one would ask: What is the chemical class of the molecule, and, accordingly, what is its expected biosynthetic class? For some chemical classes, there can be multiple biosynthetic options, e.g. peptides can be made in either a ribosomal or nonribosomal fashion. Second, one would ask: What can we specifically predict about the biosynthetic pathway? If it concerns a potential nonribosomal peptide or polyketide, knowledge of the structure would allow predicting the number of modules expected in corresponding NRPSs or PKSs, as well as their substrate specificities. Third, is there specific chemistry seen in the molecule for which enzymatic mechanisms are known? If, for example, a peptide is acylated, one could expect the presence of either a CoA-ligase or a Condensation-starter domain in the BGC. Fourth, are any other organisms known to produce this molecule? If so, one could see which BGCs have homologous clusters in each of these known producers.

When dealing with larger numbers of genomes, the abovementioned strategies may no longer be feasible. In this case, a targeted search could be done using software like clusterTools [63] or MultiGeneBlast [64] among the entire set of BGCs identified in all genomes. For example, if the presence of a certain (combination of) specific gene(s) is either desired (in case of hunting for new molecules) or expected (in case of trying to connect a known molecule to its BGC), a specific query can be built to search for this.

## Perspectives

With the recent progress in sequencing technologies and the availability of easy-to-use software programs, genome mining for BGCs and evaluating the genetic potential of secondary metabolite producing organisms have matured into an important technology. It complements the classical organic chemistry-centered approach to find, dereplicate and characterize novel bioactive secondary metabolites, and contributes toward the current paradigm-shift that brings natural products once more into focus for future drug discovery [36]. In addition, it also can be used as an effective method to evaluate the safety of biotechnological production organisms, which are used directly in food production or for the production of enzymes or other biochemicals. In this case, genome mining data can be used to demonstrate that a production strain does not contain BGCs coding for the biosynthesis of known hazardous chemicals.

Increasingly available high-quality genome data, in combination with databases of BGCs of known function, such as sequence data from the MIBiG repository [17], can also be used for dereplication of known or closely related compounds and the identification of unexplored or underexplored gene cluster families. So far, several studies [35, 49, 65–67] have successfully used such approaches to identify novel natural products. In connection with large-scale metabolomics approaches (in which gene cluster data are automatically correlated with information on known or unknown compounds identified by mass spectrometry [14, 15, 67, 68]), these high-quality data now allow for new high-throughput methods to identify novel compounds.

Many of the current limitations of automated genome mining approaches are being actively addressed by the international natural product community. The EvoMining strategy has been successfully used [50] to identify new BGCs coding for previously unknown compounds and enzymes. Another promising approach to better predict BGC boundaries is based on comparative genomics by detecting ‘breaks’ in the conserved synteny of related strains; as such breaks are often caused by the insertion and/or horizontal acquisition of BGCs, this approach allows the identification of potential secondary metabolite biosynthetic pathways without relying on previous knowledge of the enzymes involved (SYNTERUPTOR, S. Lautru and J. L. Pernodet; personal communication). Thousands of BGCs already have been identified and the number is still steadily increasing. Tools like CORASON (F. Barona-Gómez, personal communication; https://github.com/nselem/EvoDivMet; as used in [69, 70]), clusterTools [63] and MultiGeneBlast [64] can be used to identify clusters, which share varying degrees of similarity with known BGCs. Large-scale clustering of these BGCs is emerging as an important method to compare, classify into gene cluster families, dereplicate and identify novel or—depending on the aim of the study—related BGCs [49, 66, 67]. Novel software packages like BIG-SCAPE (Medema, personal communication; https://git.wageningenur.nl/medema-group/BiG-SCAPE) will help scientists to perform such analyses.

Of course, the widespread use of genome mining approaches also raises new challenges. One major bottleneck in such approaches is the frequent observation that the BGCs remain unexpressed (i.e. ‘silent’) in the producer strains under normal laboratory fermentation conditions; in such cases, the compounds cannot be detected or isolated despite the genome containing all the genes required for the biosynthesis. Thus, strategies have to be developed and improved to trigger the expression of such silent BGCs [71, 72]. One important step forward in this regard has been the development of CRISPR-based genome editing tools for important groups of bacterial and fungal secondary metabolite producers [11, 73–75] that can be used to insert promoters to activate the silent BGCs [76] or to ‘repair’ biosynthetic genes [77]. Successful expression of the BGC and isolation of a novel compound should be followed by metabolomics analysis and metabolic engineering that are interconnected with each other. Metabolomics helps with identifying secondary metabolite precursors, and hence provides clues on the use of metabolic pathways. This information in turn facilitates metabolic engineering of the host strain that considers quantitatively optimal production of a target secondary metabolite [78].

Key Points

- Despite the huge chemical diversity of bioactive secondary metabolites, the enzymes involved in their biosynthesis are often strikingly conserved.
- The sequence conservation of these enzymes can be exploited by genome mining approaches to identify secondary metabolite BGCs in genome data.
- Genome mining is a powerful method to access the genetic potential of secondary metabolite producers.
- User-friendly pipelines (e.g. antiSMASH) are available to assist scientists in genome mining.
- There are caveats that should be considered when designing and interpreting genome mining studies.
